# A Skeletal Muscle-Mediated Anticontractile Response on Vascular Tone: Unraveling the Lactate-AMPK-NOS1 Pathway in Femoral Arteries

**DOI:** 10.1093/function/zqae042

**Published:** 2024-09-17

**Authors:** Milene T Fontes, Tiago J Costa, Ricardo B de Paula, Fênix A Araújo, Paula R Barros, Paul Townsend, Landon Butler, Kandy T Velazquez, Fiona Hollis, Gisele F Bomfim, Joshua T Butcher, Cameron G McCarthy, Camilla F Wenceslau

**Affiliations:** Cardiovascular Translational Research Center, Department of Cell Biology and Anatomy, University of South Carolina, Columbia (SC) 29209, USA; Cardiovascular Translational Research Center, Department of Cell Biology and Anatomy, University of South Carolina, Columbia (SC) 29209, USA; Department of Pharmacology, Institute of Biomedical Science, University Of Sao Paulo, Sao Paulo (SP) 05508, Brazil; Cardiovascular Translational Research Center, Department of Cell Biology and Anatomy, University of South Carolina, Columbia (SC) 29209, USA; Cardiovascular Translational Research Center, Department of Cell Biology and Anatomy, University of South Carolina, Columbia (SC) 29209, USA; Cardiovascular Translational Research Center, Department of Cell Biology and Anatomy, University of South Carolina, Columbia (SC) 29209, USA; Cardiovascular Translational Research Center, Department of Cell Biology and Anatomy, University of South Carolina, Columbia (SC) 29209, USA; Department of Physiological Sciences, College of Veterinary Medicine, Oklahoma State University, Stillwater (OK) 74078, USA; Department of Pathology, Microbiology and Immunology, University of South Carolina School of Medicine, Columbia (SC) 29209, USA; Columbia Department of Veterans Affairs Health Care System, Columbia (SC) 29209, USA; Columbia Department of Veterans Affairs Health Care System, Columbia (SC) 29209, USA; Department of Pharmacology, Physiology and Neuroscience, University of South Carolina School of Medicine, Columbia (SC) 29209, USA; Cardiovascular Translational Research Center, Department of Cell Biology and Anatomy, University of South Carolina, Columbia (SC) 29209, USA; Health Research and Education Center, Federal University of Mato Grosso, Sinop (MT) 78556, Brazil; Department of Physiological Sciences, College of Veterinary Medicine, Oklahoma State University, Stillwater (OK) 74078, USA; Cardiovascular Translational Research Center, Department of Cell Biology and Anatomy, University of South Carolina, Columbia (SC) 29209, USA; Biomedical Engineering Program, University of South Carolina, Columbia (SC) 29208, USA; Cardiovascular Translational Research Center, Department of Cell Biology and Anatomy, University of South Carolina, Columbia (SC) 29209, USA; Biomedical Engineering Program, University of South Carolina, Columbia (SC) 29208, USA

**Keywords:** skeletal muscle, anticontractile response, vascular function, nitric oxide synthase, nitric oxide, lactate

## Abstract

The regulation of vascular tone by perivascular tissues is a complex interplay of various paracrine factors. Here, we investigate the anti-contractile effect of skeletal muscle surrounding the femoral and carotid arteries and its underlying mechanisms. Using male and female Wistar rats, we demonstrated that serotonin, phenylephrine, and U-46619 induced a concentration-dependent vasoconstrictor response in femoral artery rings. Interestingly, this response was diminished in the presence of surrounding femoral skeletal muscle, irrespective of sex. No anti-contractile effect was observed when the carotid artery was exposed to its surrounding skeletal muscle. The observed effect in the femoral artery persisted even in the absence of endothelium and when the muscle was detached from the artery. Furthermore, the skeletal muscle surrounding the femoral artery was able to promote an anti-contractile effect in three other vascular beds (basilar, mesenteric, and carotid arteries). Using inhibitors of lactate dehydrogenase and the 1/4 monocarboxylate transporter, we confirmed the involvement of lactate, as both inhibitors were able to abolish the anti-contractile effect. However, lactate did not directly promote vasodilation; rather, it exerted its effect by activating 5′ AMP-activated protein kinase (AMPK) and neuronal nitric oxide synthase (NOS1) in the skeletal muscle. Accordingly, Nω-propyl l-arginine, a specific inhibitor of NOS1, prevented the anti-contractile effect, as well as lactate-induced phosphorylation of NOS1 at the stimulatory serine site (1417) in primary skeletal muscle cells. Phosphorylation of NOS1 was reduced in the presence of Bay-3827, a selective AMPK inhibitor. In conclusion, femoral artery-associated skeletal muscle is a potent paracrine and endocrine organ that influences vascular tone in both sexes. Mechanistically, the anti-contractile effect involves muscle fiber type and/or its anatomical location but not the type of artery or its related vascular endothelium. Finally, the femoral artery anti-contractile effect is mediated by the lactate-AMPK-phospho-NOS1^Ser1417^-NO signaling axis.

## Graphical Abstract

**Graphical Abstract:**
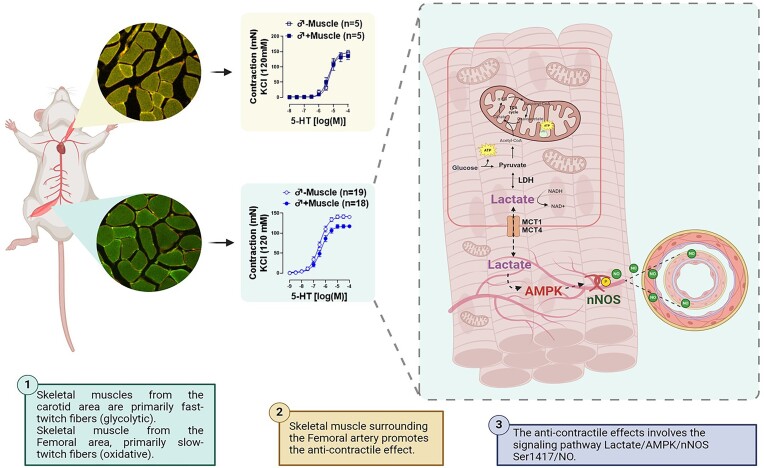
Illustration of skeletal muscle alters vascular tone. This illustration summarizes the main results obtained in the present study. Lactate released by skeletal muscle promotes AMPK activation and then the phosphorylation of NOS1 via Ser^1417^, releasing NO and thus promoting the anti-contractile effect. LDH: Lactate Dehydrogenase, MCT 1/4: monocarboxylate transporters 1 and 4, NOS1: neuronal nitric oxide synthase.

## Introduction

Maintaining adequate blood flow is essential for the functioning of all physiological systems in the body. Skeletal muscle accounts for approximately 40% of total body weight and is an organ with high metabolic demands. Therefore, the synergism between the skeletal muscle and the cardiovascular system is crucial for balancing the demand and supply of gases, nutrients, and ions necessary for maintaining physiological processes.^1^ Physiological adjustments to meet all demands during physical exercise have been extensively studied.^2–4^ However, whether skeletal muscle can regulate moment-to-moment blood flow under resting conditions is unknown.

Research into the role of perivascular adipose tissue (PVAT) has been intensely studied in recent years, emphasizing the crucial role of this tissue in regulating vascular function and mechanics.^5–9^ Specifically, PVAT releases several vasoactive substances, including nitric oxide (NO), hydrogen peroxide (H_2_O_2_), adiponectin, and others, promoting an anti-contractile effect in healthy conditions.^5–8^ The anti-contractile effects of PVAT are present in almost all vascular beds where adipose tissue surrounds the vessels. However, the vasoactive factors released by PVAT differ among the vessels and depend on the type of fat composing the PVAT.^9^,^10^

Skeletal muscle is considered a paracrine and endocrine organ that can release cytokines, peptides, and some vasoactive substances that influence the metabolism and function of the other tissues it surrounds; these factors are known as myokines.^11^,^12^ Therefore, we questioned whether skeletal muscle would exert an anti-contractile effect, similar to that of PVAT. To approach this question, we selected two conduit arteries, each surrounded by distinct skeletal muscle fiber types and located in different regions: the carotid and femoral arteries. These arteries present an insignificant amount of PVAT and are surrounded predominantly by the sternohyoid and adductor muscles, respectively. With that, we hypothesized that skeletal muscle mediates an anti-contractile effect on vascular tone, and this response would depend on the type of surrounding skeletal muscle.

## Materials and Methods

### Animals

The Animal Care and Use Committee at the University of South Carolina School of Medicine approved all animal procedures and protocols used for animal experimentation (IACUC# 2595-101693-041122). The procedures followed the National Institutes of Health Guide for the Care and Use of Laboratory Animals and Animal Research Reporting of *in vivo* Experiments (ARRIVE) guidelines. Experiments were conducted on 3-mo-old male and female Wistar rats, obtained from Charles River Laboratories. Rats were maintained on a 12-h light cycle with *ad libitum* access to water and a standard chow diet (0.3% NaCl, Harlan Teklad diet TD 7034; Madison, WI, USA).

### Vascular Reactivity

Rats were anesthetized with 5% isoflurane (1 L/min 100% oxygen), and following the loss of their righting reflex, were killed by exsanguination. The femoral and carotid arteries, with their respective skeletal muscles, were then removed. In the absence or presence of their respective skeletal muscle, 2 mm rings were mounted onto DMT wire myographs (Danish MyoTech, Aarhus, Denmark) and kept in a Krebs solution (composition in mm: 118 NaCl, 24.9 NaHCO3, 4.7 KCl, 1.2 MgSO4.7H2O, 2.5 CaCl2, 1.2 KH2PO4, 5.6 glucose, and 0.026 Na2-EDTA). As previously described,^13^ both arteries were normalized to their optimal lumen diameter for active tension development. Arteries were initially contracted using 120 mmol/L potassium chloride (KCl) to test vascular smooth muscle cell integrity. Serotonin concentration-response curves (5-HT; 1 nmol/L to 100 µmol/L) were performed in the carotid and femoral arteries. Due to the carotid artery’s lack of anti-contractile effect, the remaining mechanistic experiments were conducted only on the femoral artery. To verify whether this anti-contractile effect was receptor-dependent, two additional agonists were tested, phenylephrine (Phe; 1 nmol/L to 10 µmol/L) and thromboxane A_2_ mimic (U-46619, 0.1 nmol/L to 10 µmol/L). To assess whether the physical attachment between the artery and the muscle was necessary for the anti-contractile effect, experiments were carried out with attached or detached skeletal muscle. For detached skeletal muscles, isolated muscles were placed and positioned near the artery inside the DMT chamber. With that, we could understand whether the role of the skeletal muscle was due to contact (e.g., innervation) and/or to factors released by the skeletal muscle (see illustration in Figure 2D). Subsequently, to evaluate whether the carotid and femoral responses were due to differences between the muscle type or the type of artery, the carotid, basilar, and superior mesenteric arteries were mounted in the presence of the femoral skeletal muscle. Concentration-response curves were performed using the agonists (Phe 1 nmol/L to 10 µmol/L) or (serotonin, 5-HT; 10 pmol/L to 1 µmol/L).To begin elucidating the involved mechanism, femoral arteries with attached skeletal muscles were incubated for 30 min prior to their concentration response curves using the following inhibitors: the non-specific NOS inhibitor (N^G^-nitro-l-arginine methyl ester, 100 µm), the neuronal nitric oxide synthase inhibitor (*N*-propyl-l-arginine, 2 µm), the lactate dehydrogenase inhibitor (GSK2837808A, 10 µm), and the monocarboxylic acid transport inhibitor (2-Cyano-3-(4-hydroxyphenyl)-2-propenoic acid, 1 mm) Furthermore, to evaluate the involvement of potassium channels, we used specific blockers for the following channels: IK_Ca_ (TRAM-34 10 µm), SK_Ca_ (ULC1684, 100 nm), K_ATP_ (Glibencamide, 1µm) and BK_Ca_ (Iberiotoxin 100 nm).

### Lactate Assay

The skeletal muscles surrounding the femoral and carotid arteries (10 mg) were removed and the lactate concentration was measured following the manufacturer’s recommendations (ab65331, Abcam). All samples were deproteinized with the Deproteinizing Sample Preparation Kit-TCA (ab204708, Abcam). The protein concentration was measured using the BCA protein assay kit (ThermoFisher Scientific, 23 227) and was used to normalize the lactate values obtained.

### RT q-PCR

According to the manufacturer’s instructions, total RNA was extracted from skeletal muscle using TRIzol reagent (Invitrogen.; #15596026). cDNA was synthesized from 50 ng of the total extracted RNA using the qPCR-SuperMix-UDG Kit (Bio-Rad #1708891). Quantitative RT-qPCR was performed using the SYBR Green PCR kit (Bio-Rad; #1708882) to amplify genes of interest following the specific primers listed (Table 1). Cycle threshold (Ct) values were obtained for each gene. The difference was assigned as ΔΔCt. The fold change between the two samples was then calculated as 2−ΔΔCt, a value directly proportional to the copy number of complementary DNA and the initial quantity of mRNA. The analysis of the mRNA of interest was normalized to glyceraldehyde-3-phosphate dehydrogenase (GAPDH) and the data were expressed as the fold of change in relative to male femoral data.

**Table 1.** Genes and Primers Sequence

Gene	Primer sequence (5′-3′)
NOS1	F: GAACACGTTTGGGGTTCAGC R: CTGAGATGATCACGGGAGGC
MyH7	F: GAGACGGACGCCATACAGAG R: CCTCTGCTTCTTGTCCAGGG
MyH2	F: CCCTCAGAGAGAGCAGAGGT R: TVTAGGAGCCCCAGAAGACC
MyH1	F: CGGTCGAAGTTGCATCCCTA R: TTACAGTAGCGCCACCTTCG
MyH4	F: AGAGAGGAGCAGGAGAGTGG R: TGTCCTCCATCTCTCCCTGG
GAPDH	F: TGTTCCAGTATGACTCTACC R: GGGAGTTGTCATATTTCTCG

### Primary Skeletal Muscle Cells

The skeletal muscles surrounding the carotid (sternohyoid) and femoral (adductors) arteries were removed and cleaned of connective and adipose tissues using sterile surgical instruments. The muscles were cut into small segments and incubated in an enzymatic digestion solution [collagenase II (500 U/mL), collagenase D (1.5 U/mL), dispase II (2.5 U/mL), and CaCl2 (2.5 mm)] for 60 min in 37°C water bath, while the tube agitated every 5 min. Afterward, the solution was centrifuged (1100 × *g* for 5 min) and resuspended in a differentiation medium (high glucose DMEM, 10% horse serum, 10% fetal bovine serum, and 1% penicillin-streptomycin-glutamine). Cells were placed in flasks pretreated with matrigel, and incubated at 37°C, with 5% CO_2_. The growth medium was changed every 2 days, and when the plates became confluent, the cells were transferred to larger plates. All procedures followed pre-established protocols for skeletal muscle cell isolation.^14^ At the third passage, the cells were placed in 6-well plates and treated with Lactate (5 mm), Lactate plus Bay 3827 (selective AMPK inhibitor; 5 μm), or vehicle for 1 h.

### Immunofluorescence Analysis

Samples from skeletal muscles surrounding the carotid (sternohyoid) and femoral (adductors) arteries were washed briefly in ice-cold PBS and fixed in fresh 4% paraformaldehyde (Thermo Scientific, J19943-K2) at 4°C for 24 h. Subsequently, the tissues were immersed in 15% sucrose (S5-500, Fisher Scientific) for 12 h, followed by 30% sucrose overnight. Subsequently, the tissues were frozen in Tissue-Tek^®^ O.C.T. Compound (4583, Sakura Finetek) and cut into 6 μm thick sections using a cryostat (HM525 NX, Thermo Fisher Scientific) maintained at −20°C.^15^ All primary antibodies for this experiment were purchased from the Developmental Studies Hybridoma Bank (University of Iowa, USA), and secondary antibodies were purchased from Invitrogen, and Thermo Fisher Scientific. Briefly, slides were blocked and permeabilized for 2 h in PBS (0.01 M) containing 1% bovine serum albumin and 0.5% Triton X-100 at room temperature. Next, slides were incubated with primary antibodies against Laminin (2E8 mouse IgG2a, 1:50), MHC I (BA-F8 mouse IgG2b; 1:12,5), and MHC IIB (BF-F3 mouse IgM; 1:25) overnight at 4°C. Afterward, slides were washed 3 times in PBS for 5 min and then incubated with secondary antibodies for Laminin (goat anti-mouse IgG2a, Alexa Fluor 546; 1:500); MHC I (goat anti-mouse IgG2b, Alexa Fluor 488; 1:500); MHC IIb (goat anti-mouse IgM Alexa Fluor 594; 1:500) for 90 min at room temperature. Slides were washed 3 times in PBS for 5 min and mounted between the slide and coverslip using Fluoroshield with DAPI mounting medium (F6057, Sigma). Image acquisition was performed using the Leica Stellaris 5 confocal microscope from the Instrumentation Resource Facility at the University of South Carolina, using a 20× objective lens. Fluorescence images for each immunohistochemistry marker were obtained using the same acquisition settings (laser power and gain) for slides/skeletal muscle from different groups.

### Mitochondrial Respirometry

In another set of experiments, dissected skeletal muscle was used to evaluate mitochondrial respiration via high-resolution respirometry. The samples were rapidly weighed and placed in ice-cold biopsy preservation solution (BIOPS, 2.8 mm CaK_2_EGTA, 7.2 mm K_2_EGTA, 5.8 mm ATP, 6.6 mm MgCl_2_, 20 mm taurine, 15  mm sodium phosphocreatine, 20  mm imidazole, 0.5 mm dithiothreitol and 50 mm MES, pH = 7.1).^16^ Subsequently, the muscles were permeabilized in a BIOPS solution with 25 μg/mL of saponin at 4°C with gentle agitation for 30 min.^17^ After that, the tissues were washed for 10 min with ice-cold MiR05 (0.5 mm EGTA, 3 mm MgCl_2_, 60 mm potassium lactobionate, 20 mm taurine, 10 mm KH_2_PO_4_, 20 mm HEPES, 110 mm sucrose, and 0.1% (w/v) BSA, pH = 7.1), and quickly weighed on a precision balance, before starting the protocol. Approximately 10-15 mg was used to measure mitochondrial respiration rates at 37°C using high-resolution respirometry (Oroboros Oxygraph 2 K, Oroboros Instruments, Innsbruck, Austria). Respiration due to oxidative phosphorylation was measured using different substrates to activate specific complexes. Initially, malate (2 mm), pyruvate (10 mm), and glutamate (20 mm) were added together. To this solution, ADP (5 mm, complex I activity) was added; at the plateau of this response, succinate (10 mm; complex II) was added. The maximum capacity of the electron transport system (ETS) was assessed using the carbonyl cyanide m-chlorophenyl hydrazone (CCCP; successive titrations of 0.2 μm until maximum respiratory rates were reached). Oxygen consumption in the uncoupled state due to complex II activity was measured by inhibiting complex I by adding rotenone (0.1 μm; ETS CII). Electron transport through complex III was inhibited by the addition of antimycin (2 μm) to obtain residual oxygen consumption (ROX) levels due to oxidizing side reactions outside mitochondrial respiration. The O_2_ flow obtained at each step of the protocol was normalized by the wet weight of the tissue sample used for analysis and corrected for ROX.^18^

### Western Blotting

Samples from skeletal muscles surrounding the carotid (sternohyoid) and femoral (adductors) arteries and skeletal muscle cells (as described above) were homogenized and lysed in lysis buffer (cOmplete Lysis-M, Rocher, Mannheim, Germany) containing protease and phosphatase inhibitor cocktail (cOmplete Tablets, Roche, Mannheim, Germany). Samples were centrifuged (13 000 × *g* for 15 min at 4°C), and supernatants were isolated and stored at −80°C. Protein concentration was subsequently determined using the BCA method, and then equal quantities of protein (50 μg) were loaded into 10% and 8% polyacrylamide gels. After loading, gels were separated by sodium dodecyl sulfate-polyacrylamide gel electrophoresis and transferred to 0.45-μm Amersham Protran nitrocellulose membranes (GE Healthcare). Antibodies used: anti-NOS1 (1:1000; Thermo Fisher Scientific #37-2800), anti-NOS1 phospho S847 antibody (1:1000; abcam, ab16650), anti-NOS1 phospho S1417 antibody (1:1000; abcam, ab5583), superoxide dismutase 3/extracellular (EC)-SOD antibody (1:5000, abcam, ab8318), and superoxide dismutase 2/manganese (MnSOD) antibody (1:5000, Cell signaling, 13 141s). Anti-GAPDH (1:10 000; Abclonal, ac001) was used as loading control antibodies. Membranes were incubated with the matched secondary antibody (1:5000) at room temperature for 90 min. The blots were scanned using a Gene Gnome Bioimaging system (Syngene). Image J [National Institutes of Health and the Laboratory for Optical and Computational Instrumentation (LOCI, University of Wisconsin)] was used to quantify the scanned images.

### Co-immunoprecipitation

Co-immunoprecipitation was performed using a modified Thermo Scientific Pierce Kit (Thermo Fisher Scientific, Rockford, IL, USA). Fibers from adductor skeletal muscles were lysed and total protein was extracted via pulverization of frozen tissue following sonication (3×, 10 s, Setting 5) in extraction RIPA buffer [1% v/v NP-40 in Tris-buffered saline (TBS; 50 mm Tris-HCl, pH 7.5, 150 mm NaCl)] with 0.5 mm PMSF, PIC1, PIC2, 500 nm 5 mm NaF, and 5 mm β-glycerophosphate (or with phosphatase and protease inhibitors). Tissue debris was pelleted at 10 000 × *g* (30 min at 4°C) and protein concentration was estimated using the BCA. Five hundred microgram of protein per 1 μg of antibody (NOS1 #4231, cell signaling,—concentration 200 μg/mL) was used to form a protein complex, and allowed to immunoprecipitate for 24 h. Thermo Scientific Protein A/G was used to capture the antibody: protein complex for 2 h at 4°C. Immunoprecipitated columns were washed with extraction buffer to remove non-specifically bound proteins, and then resuspended in 2× SDS buffer with 350 mm DTT, and heated at 95°C for 5 min. An equivalent amount of rabbit IgG antibody was used as an immunoprecipitation control. The immunoblots were developed using AMPK antibody (1:1000, cell signaling, #2532) for 24 h at room temperature or overnight at 4°C. Antigens were detected using the Pierce ECL Western Blotting Substrate (Thermo Scientific, IL, USA). Direct Blue staining was used as a loading control staining method.

### Data and Statistical Analysis

Sample sizes are described in the graphs, with each dot representing an independent rat. All data are presented as mean ± SEM. Statistical analyses were performed using GraphPad Prism software version 10.2.3 9. The Shapiro–Wilk test was used to check the normality of the data. Depending on the data distribution and experimental design, Student’s *t*-test, 1- or 2-way ANOVA was used as appropriate, followed by the Tukey post hoc test. A value of *P *< 0.05 was considered statistically significant.

## Results

### Skeletal Muscle Surrounding the Femoral Artery Exhibits an Anti-contractile Effect, Unlike the Carotid Artery, and Is Independent of Sex

**Figure 1.**
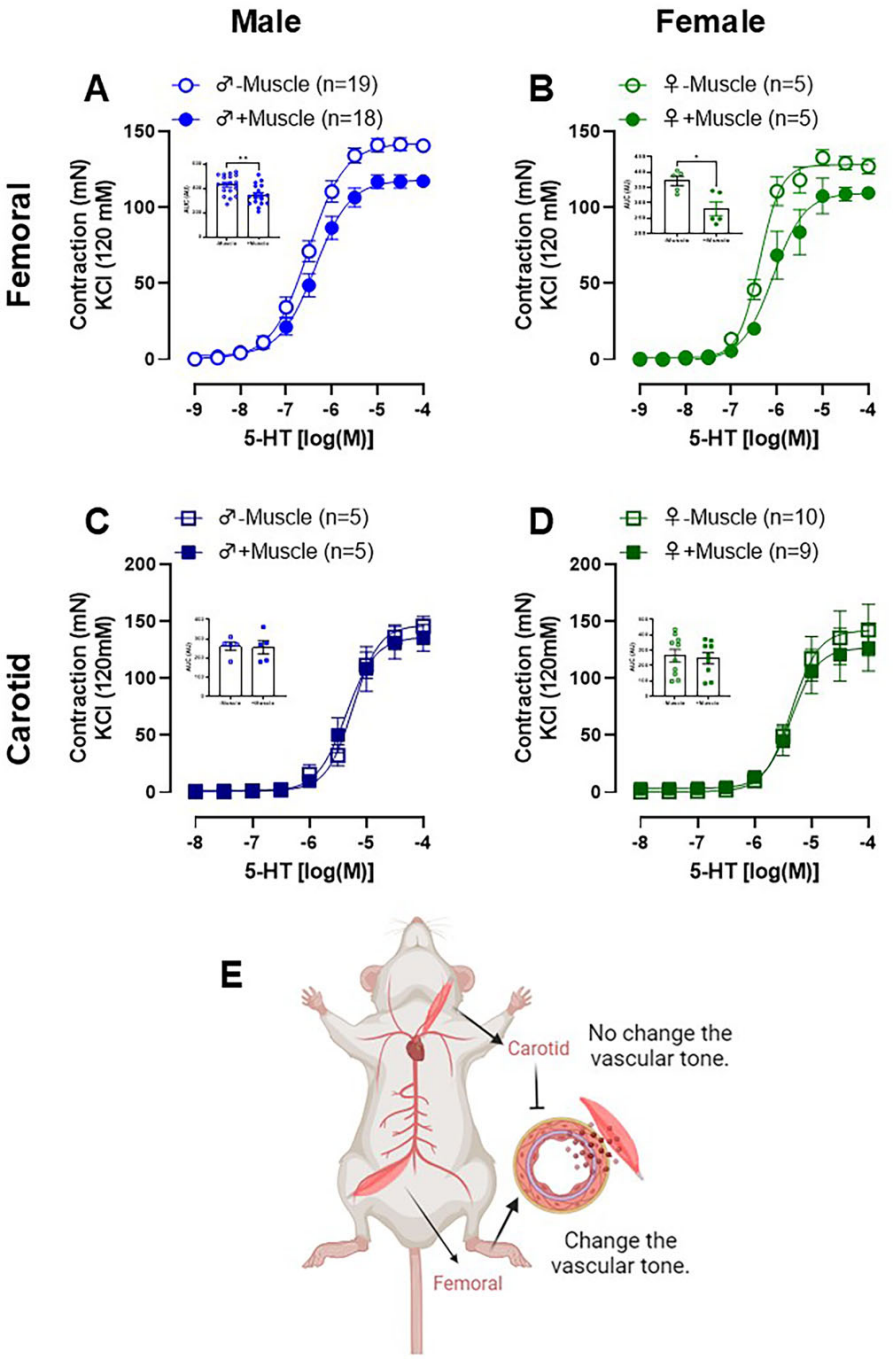
Serotonin (5-HT)-induced contraction in femoral (A and B) and carotid (C and D) arteries rings with functional endothelium in the presence (+muscle) or absence (−muscle) of respective skeletal muscle tissue from male (A and C) and female (B and D) Wistar animals. The figure bar graph represents the area under the curve (AUC) to 5-HT in the presence or absence of skeletal muscle in both arteries and sex. This figure highlights an anti-contractile effect of skeletal muscle surrounding the femoral, absent in the skeletal muscle surrounding the carotid arteries (E). The results are expressed as the mean ± SEM. The number of animals used in each experiment (*n*) is expressed in parentheses or dots. Statistic: t test (*P < 0.05; **P < 0.01).

**Figure 2.**
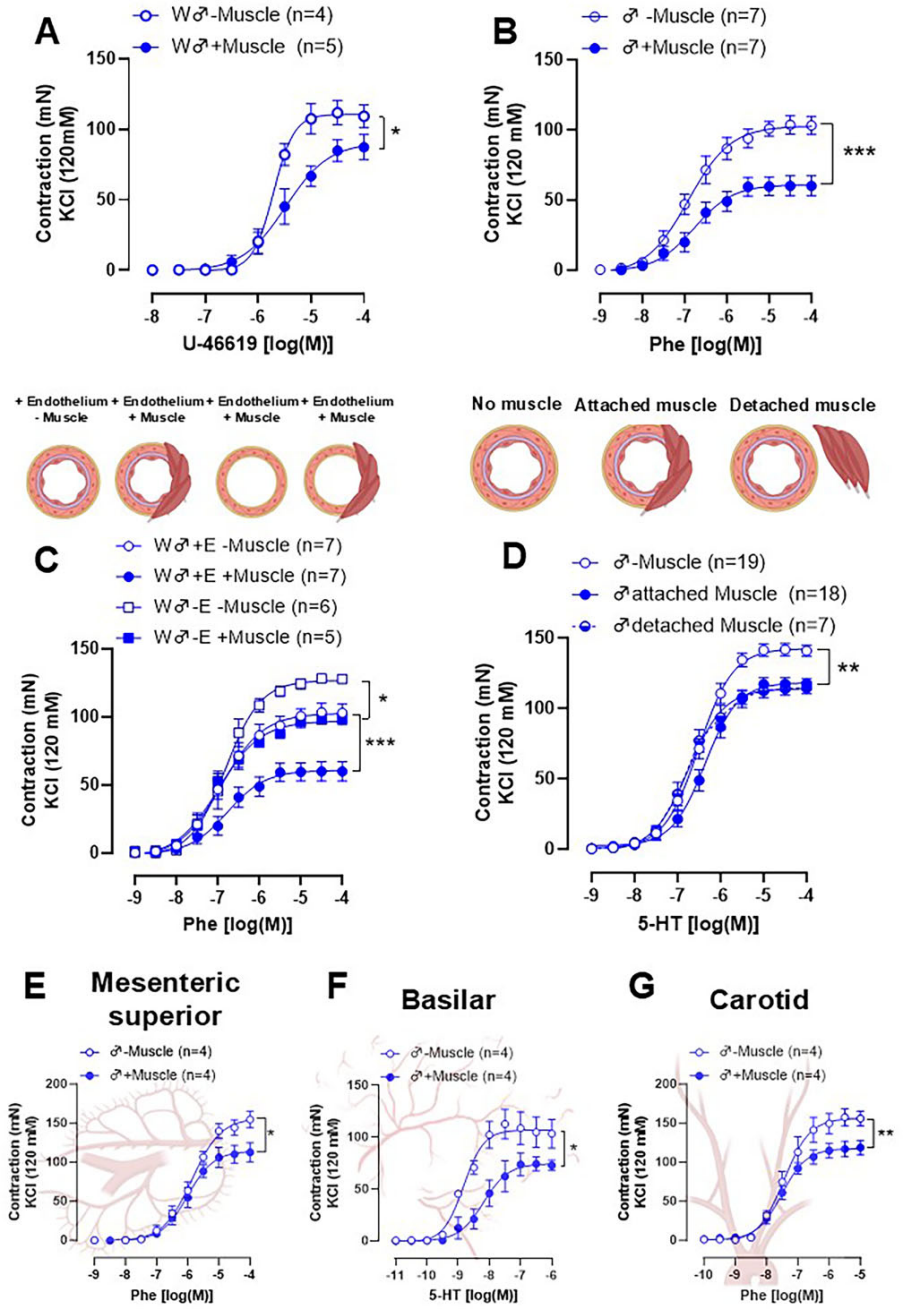
Concentration-response curves to U-46619 (A) and Phenylephrine (Phe, B) in femoral artery rings from Wistar rats in the absence (−muscle) or presence (+muscle) of the skeletal muscle. Concentration-response curves to Phenylephrine (Phe) was performed in femoral artery rings from Wistar rats in the absence (−muscle) or presence (+muscle) of the skeletal muscle with and without endothelium functional (C). Concentration-response curves to Serotonin (5-HT) was performed in rings without skeletal muscle (−muscle), with attached muscle (artery connected to the muscle), and detached muscle (poisoned near the artery in the chamber). Mesenteric superior (E), Basilar (F), and Carotid (G) were mounted in the presence of the skeletal muscle surrounding the femoral artery. The number of animals used in each experiment (*n*) is expressed in parentheses. The results are expressed as the mean ± SEM. Statistic: two-way ANOVA *P < 0.05; **P < 0.01. Please note that in some cases, the controls [presence or absence of muscle (−/+Muscle)] are consistent across all graphs, as the first myograph chamber was used as the control, while the other chambers were exposed to different inhibitors. To enhance clarity for the reader, we have presented these results in separate graphs.

As expected, serotonin promotes a concentration-dependent vasoconstrictor response in isolated femoral and carotid arteries from male and female Wistar rats. Interestingly, when the skeletal muscles surrounding the arteries were maintained to assess contractile capacity, the serotonin-induced contraction was reduced, but only in the femoral artery (Figure 1A-B), and this effect was sex independent. These data suggest that skeletal muscle surrounding the femoral artery promotes an anti-contractile effect. Importantly, this effect was also observed when using an alpha-1 adrenergic agonist (Phe) and the thromboxane A_2_-mimetic (U-46619) (Figure 2A-B), indicating this anti-contractile factor acts in a receptor-independent manner. To investigate whether this effect depends on the endothelium, a subgroup of femoral artery rings had their endothelium removed. As expected, endothelium removal (−E) increased the response to Phe in arteries absent of skeletal muscle (−Muscle). However, the presence of muscle (+Muscle) in arteries without endothelium (−E) had the same effect, suggesting that the anti-contractile effect promoted by skeletal muscle is independent of the endothelium (Figure 2C). In addition to testing the effects of skeletal muscle presence, rings were mounted in which the muscle was separated from the artery, but kept close (detached) to evaluate whether the anti-contractile effect was dependent on tissue connection. Similar to the attached muscle, the detached muscle also caused decreased contraction (Figure 2D).

### The Skeletal Muscle-Induced Anti-Contractile Effect Is Vascular Bed Dependent

**Figure 3.**
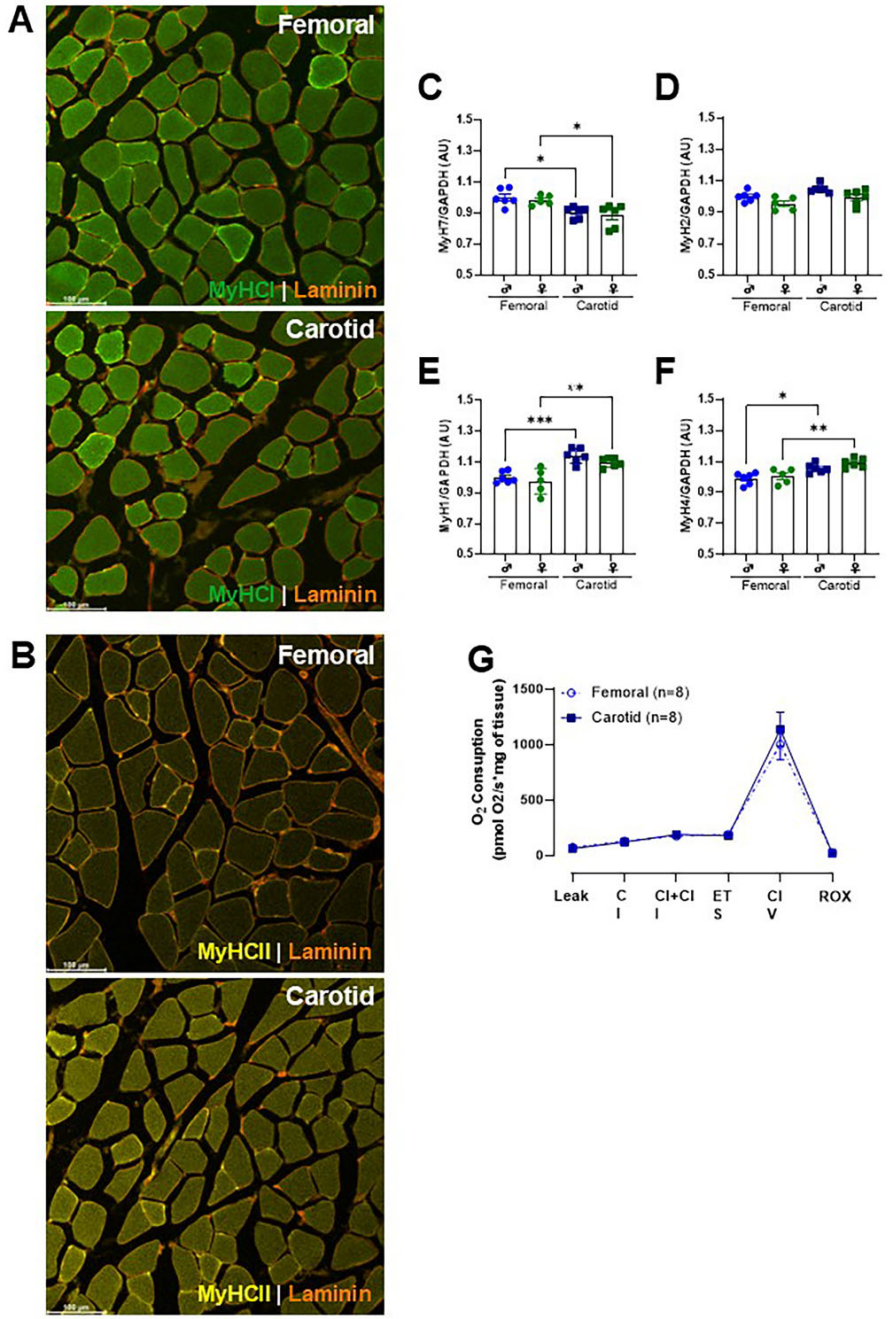
Fiber type-specific distribution of selected markers revealed by immunofluorescence analysis from skeletal muscles surrounding the carotid (sternohyoid) and femoral (adductors). Fiber types are labeled MyHC1 (type 1, green; A), MyHCII (type 2b, yellow; B), and Laminin (red). Gene expression of type I (MyH7; C), type IIa (MyH2; D), type IIx (MyH1; E), and type IIb (MyH4; F) in skeletal muscles surrounding the carotid and femoral arteries. Representative graphs show the oxygen consumption rate measurement upon adding different substrates and inhibitors in skeletal muscles surrounding the carotid and femoral arteries (G). The number of animals used in each experiment (*n*) is expressed in parentheses or dots. The results are expressed as the mean ± SEM. Statistic: 1-way ANOVA or Student’s *t*-test as appropriate, *P < 0.05; **P < 0.01; ***P < 0.001.

Since we did not observe the anti-contractile effect in skeletal muscle surrounding the carotid artery (Figure 1C-D), we then questioned whether the lack of response was due to the type of artery and/or the type of skeletal muscle. To answer this question, we mounted three arteries from distinct vascular beds, which are surrounded by different perivascular tissues [mesenteric artery (white adipose tissue), basilar artery (subarachnoid tissue), and carotid artery (skeletal muscle)], and then these arteries were placed adjacent to the skeletal muscle that surrounds femoral arteries. Of note, we used similar-sized skeletal muscle and the diameter of arteries. We observed that the anti-contractile effect occurred in all arteries evaluated, including the carotid artery, which did not show this effect in the presence of its respective muscle (Figure 2E, F, and G). Subsequently, we then evaluated the gene expression of specific markers for Type I (MyH7), Type IIa (MyH2), Type IIx (MyH1), and Type IIb (MyH4) muscle fibers in skeletal muscle from the carotid and femoral arteries. We observed that the muscle adjacent to the femoral artery has the highest gene expression for MyH7, the Type I fiber marker (Figure 3C). In contrast, the carotid muscle showed higher expression for MyH1 and MyH4, the Type IIx (Figure 3E) and IIb fiber markers (Figure 3F), respectively. There were no differences between skeletal muscles for fiber marker IIa (Figure 3D). Immunofluorescence analysis confirmed the predominance of type II fibers in the carotid artery, whereas type I fibers did not differ between the muscles evaluated (Figure 3A and B). Interestingly, we did not observe any difference between the muscles used in mitochondrial respiratory capacity (Figure 3G).

### The Anti-Contractile Effect Involves Lactate-AMPK-NOS1-NO Signaling

**Figure 4.**
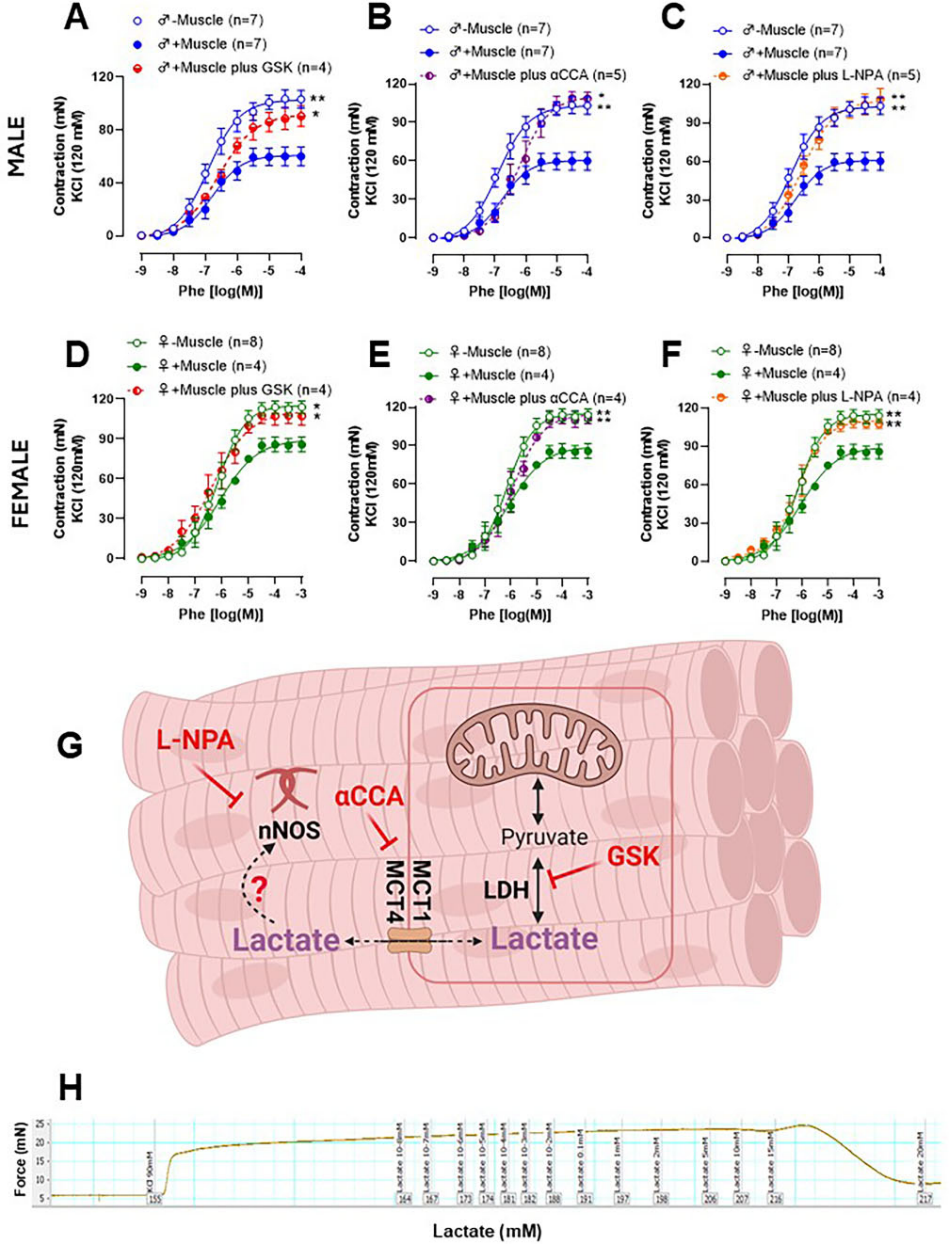
Concentration-response curves to Phenylephrine (Phe) performed in femoral artery rings from male and female Wistar rats in the absence (−muscle) or presence (+muscle) of the skeletal muscle, with the lactate dehydrogenase inhibitor [GSK2837808A, (GSK); A and D], and the monocarboxylic acid transport inhibitor [2-Cyano-3-(4-hydroxyphenyl)-2-propenoic acid (αCCA); B and E] and the neuronal nitric oxide synthase inhibitor [*N*-propyl-l-arginine (L-NPA); C and F]. Representative figure with the signaling pathways was blocked in the vascular reactivity experiment (G). Typical recording of a concentration-response curve to lactate (10 µm-25 mm) in the femoral artery (−muscle) rings pre-contracted with KCl (90 mm) (H). The number of animals used in each experiment (*n*) is expressed in parentheses. The results are expressed as the mean ± SEM. Statistic: 2-way ANOVA or Student’s *t*-test as appropriate, *P < 0.05; **P < 0.01. Please note that in some cases, the controls [presence or absence of muscle (−/+Muscle)] are consistent across all graphs, as the first myograph chamber was used as the control, while the other chambers were exposed to different inhibitors. To enhance clarity for the reader, we have presented these results in separate graphs.

Previous publications have demonstrated that lactate can promote relaxation in different vascular beds.^19–24^ Therefore, we hypothesized that lactate released from the femoral artery skeletal muscle would be responsible for the anti-contractile effect. To test this hypothesis, we pre-incubated the femoral artery rings (+Muscle) with the lactate dehydrogenase inhibitor [GSK2837808A (GSK), Figure 4A and D] and the monocarboxylate transporter inhibitor [α-cyano-4-hydroxycinnamic acid (αCCA), Figure 4B and E]. Both inhibitors abolished the anti-contractile effect seen with the skeletal muscle on the femoral artery in males and females (Figure 4A, B, D, and E). Subsequently, we performed a concentration-response curve for lactate (0.1-20 mm) in isolated femoral arteries (-Muscle) contracted with KCl or Phe. Interestingly, lactate did not promote changes in vascular tone in the physiological ranges, and we observed a loss of vascular tone at high concentrations (<15 mm, pH ∼3; Figure 4H). These data suggest that lactate cannot promote direct dilation in the femoral artery, but since its inhibitors, LDH (Figure 4A and D) and MCT1/4 (Figure 4B and E), reversed the anti-contractile effect, we then had an alternative hypothesis that lactate could promote its anti-contractile effects in an autocrine way. Specifically, we hypothesized that lactate in the skeletal muscle activates potassium channels.^25^ Therefore, we used specific potassium channel blockers including, intermediate-conductance, calcium-activated potassium channels (TRAM-34 10 µm), calcium-activated potassium channels (ULC1684, 100 nm), an ATP-sensitive potassium channel (Glibencamide, 1 µm) and large-conductance calcium-activated potassium channels (Iberiotoxin 100 nm). Interestingly, none of these inhibitors altered the anti-contractile response elicited by femoral skeletal muscle (Table 2).

**Table 2.** The difference in the area under the curve between the absence and presence of anti-contractile inhibitors

Blocker	−Muscle	+Muscle
Tram-34 + UCL 1684	−0.48 ± 3.9 (4)	−0.81 ± 5.5 (5)
Glibenclamide	−3.20 ± 9.0 (4)	9.39 ± 15.2 (5)
Iberiotoxin	19.9 ± 10.2 (6)	6.97 ± 6.3 (6)
L-NAME	25.00 ± 8.3 (5)	434.65 ± 15.8 (5)***
L-NPA	−7.71 ± 3.4 (5)	347.01 ± 31.3 (5)***

***P ≤ 0.01 versus −Muscle.

Previously, it has been shown that lactate could affect NO production in the vascular cells.^19^,^20^ Neuronal nitric oxide synthase (NOS1) is an important enzymatic source of NO in skeletal muscle. Therefore, the non-specific NOS inhibitor (L-NAME) and the specific NOS1 inhibitor (L-NPA) were used to evaluate their role in the anti-contractile effect caused by lactate. L-NPA abolished the anti-contractile effect in arteries from both sexes (Figure 4C and F). It is important to mention that L-NAME promoted the blocking of the anti-contractile effect to the same extent as inhibitor L-NPA (Table 2).

**Figure 5.**
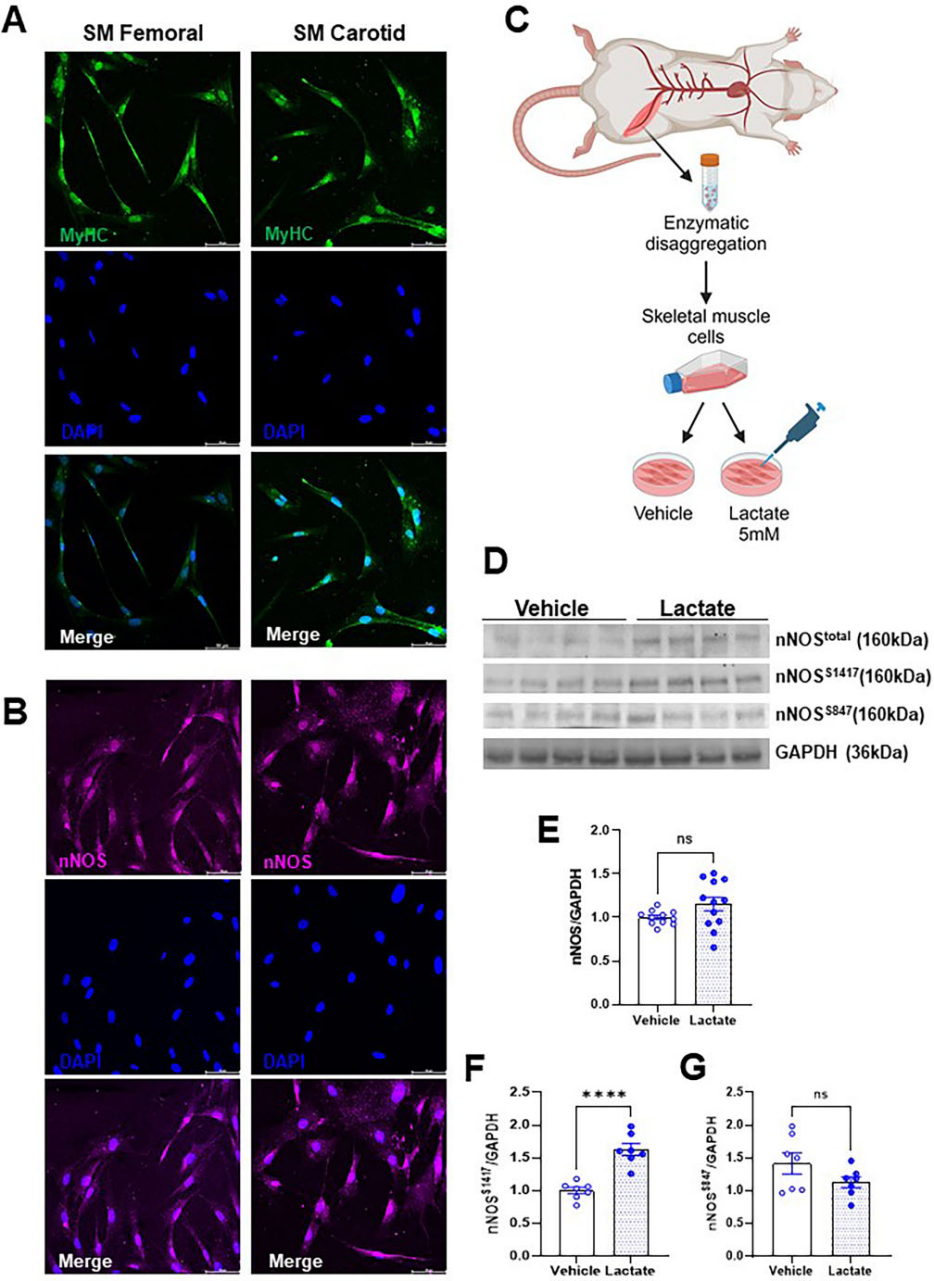
Immunofluorescence staining for MyHC (Myosin heavy chain, A) and NOS1 (neuronal nitric oxide synthase, B) in cells from skeletal muscles surrounding the carotid (sternohyoid) and femoral (adductors) arteries from Wistar rats. Representative images shown (MyHC, green; NOS1, pink; DAPI, blue), original magnification 20×. The Representative figure shows the protocol performed with skeletal muscle cells, which were isolated, cultured, and treated with lactate (5 mm, 1 h) or vehicle (C). Graphical representation of the expression of total NOS1, Ser1417, Ser847 and GAPDH as loading control (D). Relative amounts of total NOS1 (E), Ser1417 (F), and Ser847 (G) proteins were shown by densitometry. Each lane was loaded with 50 μg of total protein. The number of animals used in each experiment (n) is expressed in dots. The results are expressed as the mean ± SEM. Statistic: Student’s *t*-test, ^****^P < 0.0001. Please note that the original membrane referenced in Figure 5 are in the Suppl. Figure S1.

**Figure 6.**
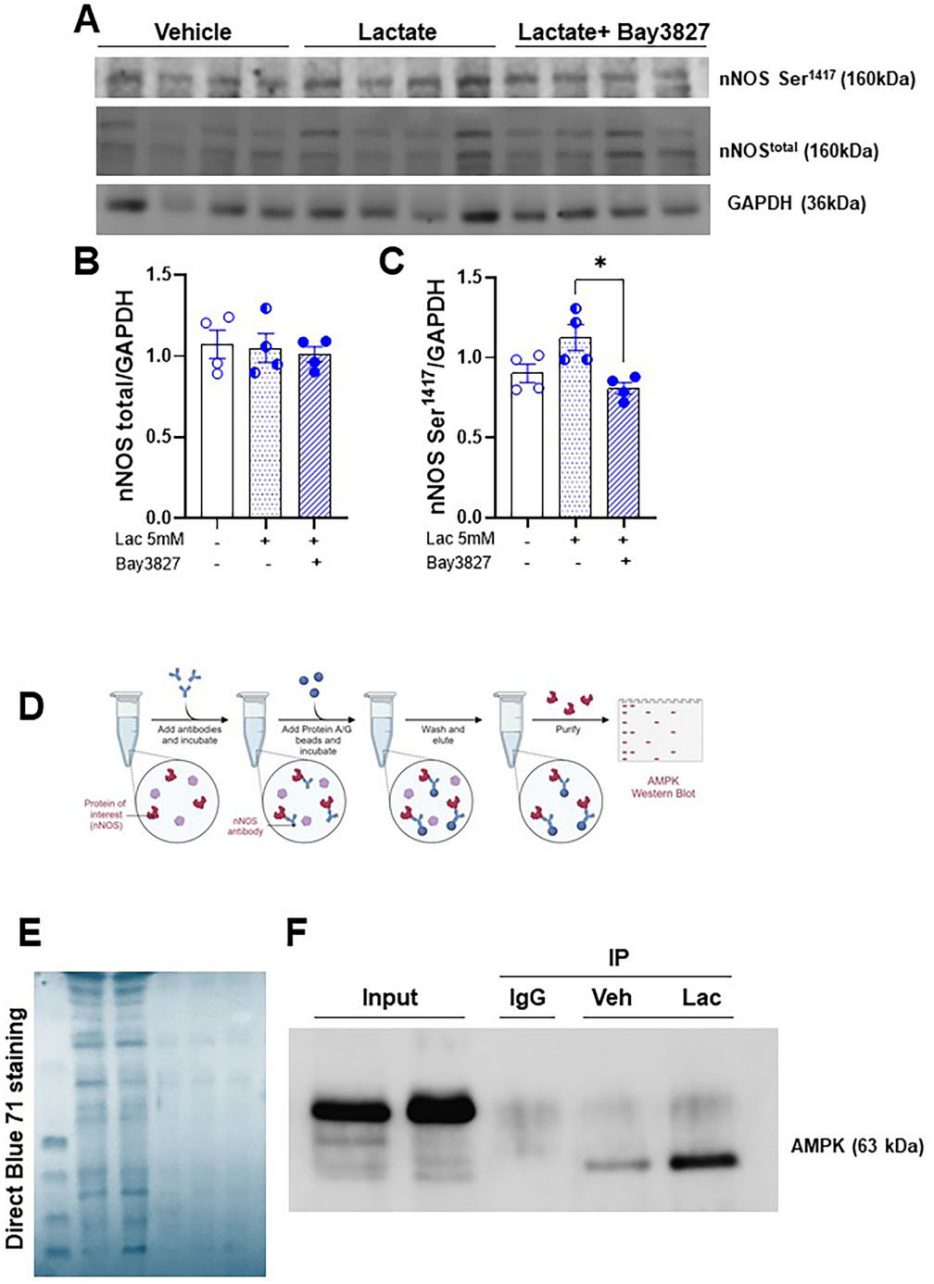
Representative western blot image of cells from the muscle that surrounds the femoral artery, treated with lactate (5 mm, 1 h) and lactate plus Bay 3827 (specific AMPK inhibitor, 5 μm) and vehicle (A). Bar graphical representation of the expression of total NOS1 (B), NOS1^Ser1417^(C) and GAPDH as loading control. Illustrative figure of co-immunoprecipitation assays showing interactions of NOS1 with AMPK (D). Coomassie blue staining was used in western blot analysis (E) as a loading control staining method. Representative western bott image for AMPK of NOS1 co-immunoprecipitation in cells treated with lactate (5 mm, 1 h) or vehicle (F). Relative amounts of proteins were shown by densitometry. Each lane was loaded with 50 μg of total protein. The number of animals used in each experiment (*n*) is expressed in dots. The results are expressed as the mean ± SEM. Statistic: 1-way ANOVA, *P < 0.05. Please note that the original membrane referenced in Figure 6 are in the Suppl. Figures S2-3.

Subsequently, we cultured primary skeletal muscle cells to corroborate these findings and for more mechanistic insights. First, we confirmed that the cells originating from the surrounding femoral and carotid artery muscles expressed myosin heavy chain, a specific marker of skeletal muscle cells (Figure 5A) and NOS1 (Figure 5B). We then treated cells isolated only from the femoral muscle with lactate (5 mm; 1 h) to evaluate NOS1 activation via changes in phosphorylation. Lactate increased NOS1 phosphorylation at Ser1417, the main post-translational modification responsible for NO release from NOS1 (Figure 5E, Suppl. Figure S1). On the other hand, no differences were observed in the phosphorylation at Ser847 (Figure 5D, G and Suppl. Figure S1). No changes in the total expression of NOS1 were observed (Figure 5D, 5E, and Suppl. Figure S1). Previous literature data demonstrated that lactate activates AMPK, and this activation is responsible for different effects such as remodeling the cellular metabolic profile, and proliferation and differentiation of cells in skeletal muscle.^26^,^27^ Activated AMPK could be responsible for phosphorylating NOS1.^27^,^29^ We treated cells with lactate in the presence and absence of the specific AMPK inhibitor (Bay 3827). As we had already verified in Figure 5D-E, the presence of lactate did not change the expression of total NOS1, nor did Bay 3827 promote any change in the total expression of NOS1 (Figure 6A-B, Suppl. Figure S2). However, the presence of Bay 3827 significantly reduced the expression of NOS1^Ser1417^(Figure 6A-C, Suppl. Figure S2). To corroborate these data, we then confirmed the role of the lactate-AMPK-phospho-NOS1^Ser1417^ signaling pathway through the immunoprecipitation experiment. For this, we immunoprecipitated NOS1 from cells treated with vehicle or lactate, and we observed that AMPK expression was increased in cells treated with lactate when compared to vehicle (Figure 6D, Suppl. Figure S3).

### Antioxidant Defense Is Higher in the Muscle Surrounding the Femoral Artery

**Figure 7.**
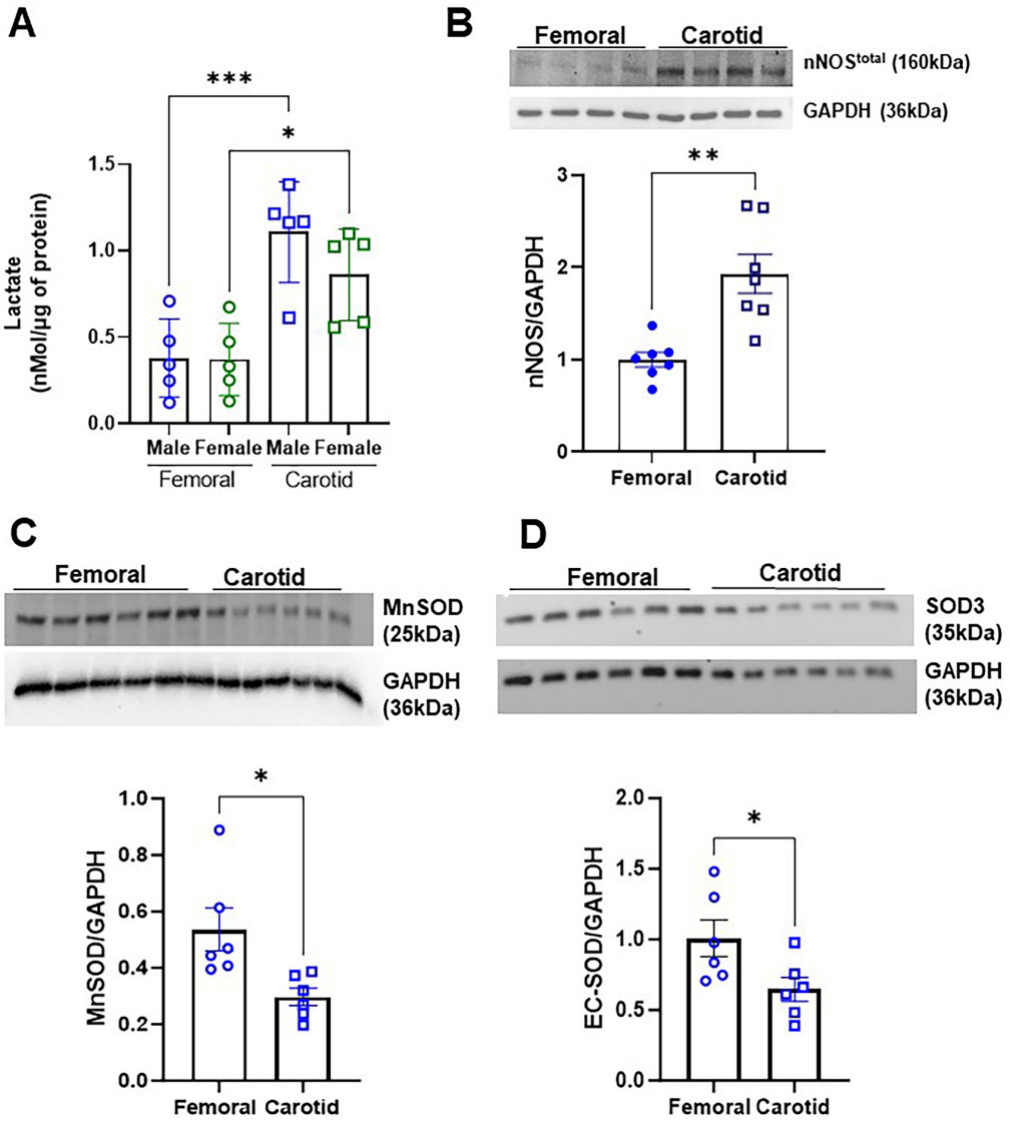
Lactate concentration of skeletal muscle skeletal muscles surrounding the carotid (sternohyoid) and femoral (adductors) of Wistar rats (A). Graphical representation and relative amounts of the expression of total NOS1 (B), manganese-dependent superoxide dismutase (MnSOD/SOD2; C), extracellular superoxide dismutase (EC-SOD/SOD3; D), and GAPDH as loading control were evaluated by densitometry. Each lane was loaded with 50 μg of total protein. The number of animals used in each experiment (*n*) is expressed in dots. The results are expressed as the mean ± SEM. Statistic: Student’s *t*-test, *P < 0.05; **P < 0.01; ***P < 0.001. Please note that the original membrane referenced in Figure 6 are in the Suppl. Figures S4-6.

Although we have observed that lactate-NOS1-NO signaling plays a major role in the skeletal muscle of the femoral arteries, when we measured the lactate concentrations and the total protein expression of NOS1 in skeletal muscle from both carotid and femoral arteries for comparison purposes, these factors were also elevated in the carotid muscle as well (Figure 7A and B), despite no evidence of the anti-contractile effect. When we evaluated the expression of two different superoxide dismutase (SOD), an important enzyme for antioxidant defense, there was a higher expression of MnSOD2 and EC-SOD3 (Figure 7C and D, Suppl. S4-6) in the muscle lining the femoral artery when compared to the carotid.

## Discussion

Muscular blood flow is controlled by several central and local mechanisms that guarantee metabolic needs are met, even under high demand.^2–4^ Evidence indicates that even at rest, skeletal muscle might release certain factors contributing to vascular tone. This concept is related to skeletal muscle as an endocrine and paracrine organ capable of secreting bioactive substances, known as myokines, into circulation.^30^,^31^ However, this concept is still controversial, and it is still unclear whether skeletal muscle modulates moment-to-moment vascular tone. Specifically, it was unknown whether skeletal muscle acts as a source of anti-contractile factors at rest, and the mechanisms by which this phenomenon would occur. Here, we show for the first time, that femoral artery skeletal muscle, but not the carotid, regardless of sex, presented with the ability to antagonize the contractile response and exert an anti-contractile effect. This effect suggests a complex interaction between vascular and perivascular tissues. Our data showed that local mechanisms mediate this response due to the *ex vivo* nature of our experiments being performed in isolated organ baths, thus removing most of the systemic and central influences. Further, the direct contact of skeletal muscle with the vasculature does not seem essential for its regulatory role, as skeletal muscle detached from the arteries did not lose its anti-contractile capacity.

An important observation was that the anti-contractile effect differed between regions and/or types of skeletal muscle, but not between types of arteries evaluated. The physiological function and composition of each tissue or type of muscle possibly play a vital role in these differences. Further, the differences between the types of skeletal muscle fibers can also play a role. Here, by using two different approaches, qPCR and immunofluorescence, we observed a mix in the types of fibers in the sternohyoid and adductor, showing that these muscles are not composed of only one type of skeletal muscle fiber. However, the proportion of fiber types differs between the skeletal muscle surrounding the femoral artery and the carotid artery. The skeletal muscle surrounding the femoral artery exhibits characteristics of type 1 fibers (slow-twitch/oxidative metabolism), whereas the muscle surrounding the carotid arteries presents a predominance of type 2 (fast-twitch/glycolytic metabolism). Supporting these findings, it has previously been demonstrated that changes in a rat’s hindlimbs’ blood flow depend on the muscles’ fiber type composition.^32^

Skeletal muscle can release several metabolic substances called myokines (a factor released by skeletal muscle).^30^,^31^ Among myokines, lactate has increasingly been explored as a signaling molecule and driver of biochemical and physiological processes, presenting autocrine, paracrine, and endocrine functions.^33^ Lactate is present in both types of muscle fibers, the type II fiber being mainly responsible for its production through the glycolytic pathway, and type I fibers responsible for its removal through oxidative metabolism (a process carried out in mitochondria). Lactate is a natural byproduct of cellular metabolism, with skeletal muscle being the main producer. Thus, the release and utilization of lactate occurs moment by moment, operating continuously as a myokine. Therefore, lactate has metabolic and signaling functions, as demonstrated previously and reinforced in this work.^30^ Lactate transports across the plasma membrane of all cells and between these different fiber types. Lactate transport is facilitated by monocarboxylate transport proteins (MCTs).^34^ In the present work, we used two different inhibitors to verify whether lactate was involved in the femoral artery skeletal muscle-induced anti-contraction. Specifically, we used the LDH inhibitor, which converts pyruvate into lactate, and the MCT1/4 blocker. Regardless of the inhibitor, the anti-contractile effect was abolished, suggesting the role of lactate as the possible relaxing factor derived from skeletal muscle. However, when we administered lactate directly to the arteries in a concentration-dependent manner, and in the absence of skeletal muscle, lactate failed to induce relaxation, except at higher concentrations (≥15 mm). At these higher concentrations, lactate becomes toxic by inducing acidosis, and resulting in tissue death (as seen in the typical trace, Figure 2H). This experiment led to the hypothesis that lactate’s anti-contractile effect may be indirect.

A few studies have suggested lactate as a vasodilator factor; however, these studies utilized perfusion experiments rather than isolated organs,^19^,^22^ making it difficult to ascertain whether lactate would have a direct and/or indirect effect on the vasculature. Therefore, based on the new evidence described in the present manuscript, lactate can activate different pathways, such as proteins or enzymes, directly responsible for promoting the anti-contractile. For instance, the literature cites the nitric oxide/cGMP pathway and potassium channels.^19^,^21–24^ Firstly, we performed a concentration-response curve for serotonin in the presence and or absence of important potassium channel blockers involved in the vascular tone, and we did not observe any significant changes (Table 2). Then, we focused on NOS, specifically the NOS1 form. Accordingly, the skeletal muscle NOS1 enzyme regulates several cellular processes, such as contraction, glucose metabolism, and blood flow regulation.^35–37^ The non-specific NOS inhibitor (L-NAME) and the specific NOS1 inhibitor (L-NPA) abolished the anticontractile effect, suggesting that NOS1-specific nitric oxide was being released by skeletal muscle. To confirm this premise, we used primary cells isolated from skeletal muscle surrounding the femoral artery and treated them with physiological lactate levels. We observed a significant increase in the phosphorylation of NOS1^Ser1417^ without altering total protein expression or the phosphorylation of NOS1^Ser847^. Phosphorylation at Ser 1417 activates NOS1 and increases NO production, while phosphorylation at Ser847 has the opposite effect.^38^,^39^ We used the same approach as before, adding the specific AMPK inhibitor to the lactate treatment. This experiment demonstrated the role of AMPK in activating NOS1^ser1417^. Therefore, we suggested that the anticontractile effect involves the lactate-AMPK-NOS1-NO signaling via increased phosphorylation of NOS1^Ser1417^.

In the present study, we also observe some intriguing data that need further investigation. For instance, total protein expression for NOS1 was more prominent in the muscle surrounding the carotid artery, which did not affect the anti-contractile response. Further, the carotid artery skeletal muscle presents predominantly fast-twitch fibers, as opposed to the femoral artery skeletal muscle which presents predominantly slow-twitch fibers. A possible explanation for this phenomenon would be the localization of NOS1. Accordingly, previous work has shown that NOS1 is localized in different compartments in the skeletal muscle, and this would affect its function.^37^ The limitation of the present work is that we did not evaluate the localization of NOS1, but the total expression of this enzyme. Further, carotid muscles presented with more lactate levels, which supports our findings that this type of muscle presents with the predominance of type 2 (fast-twitch/glycolytic metabolism). We suggest that the lactate-AMPK-NOS1-NO signaling, via increased phosphorylation of NOS1^S1417^ is more sensitive and compartmentalized in the skeletal muscle from the femoral arteries. For instance, the activity of NOS1 and NO bioavailability are important factors and are directly associated with the half-life of this molecule. Skeletal muscles from femoral arteries, although expressing increased activity of NOS1, present a more potent antioxidant defense system to maintain NO bioavailability. Superoxide dismutase (SODs) are functional antioxidant defense systems for maintaining delicate redox homeostasis; they are the first line of defense against oxygen-free radicals and, consequently, NO bioavailability.^40–42^ The expression of the mitochondrial manganese-containing SOD (MnSOD or SOD2) and the extracellular SOD (EcSOD or SOD3) are greater in the femoral artery muscle than the skeletal muscle surrounding the carotid artery. This suggests that the muscle that surrounds the femoral artery has a greater bioavailability of NO, since the muscle that surrounds the carotid artery possibly has an increased formation of peroxynitrite (ONOO^−^) and, consequently, lower NO bioavailability. In conclusion, we demonstrated that femoral artery skeletal muscle, regardless of sex, is a potent endocrine organ that maintains vascular tone. Perturbations of this tissue could lead to exacerbated vasoconstriction, resulting in vascular dysfunction, as observed in several cardiovascular diseases, including hypertension, and other diseases associated with sarcopenia, including aging and cancer. Mechanistically, we demonstrated that the anticontractile effect involves the type of fiber and/or anatomical location but not the type of artery and the presence of endothelium. Finally, we propose that this anticontractile effect is mediated by the lactate-AMPK-phospho-NOS1^Ser1417^-NO signaling axis.

## Supplementary Material

zqae042_Supplemental_File

## Data Availability

All data are available upon a request to the corresponding author.
