# Supplementary material for: A Skeletal Muscle-Mediated Anticontractile Response on Vascular Tone: Unraveling the Lactate-AMPK-NOS1 Pathway in Femoral Arteries
Source: Function (Oxf). 2024 Sep 17;5(6):zqae042. doi: 10.1093/function/zqae042 (PMC11577611; doi:10.1093/function/zqae042)
Supplement: zqae042_Supplemental_File [file zqae042_supplemental_file.pdf]

Suppl. Fig 1. The images below are the original blots referenced in Figure 5D. Please note that although Membrane 3 (total nNOS and GAPDH) was not used as a representative image, it was used to normalize (yellow square) pnNOS, which was also in Membrane 3. The first sample (arrow, control) was not considered for the quantification.

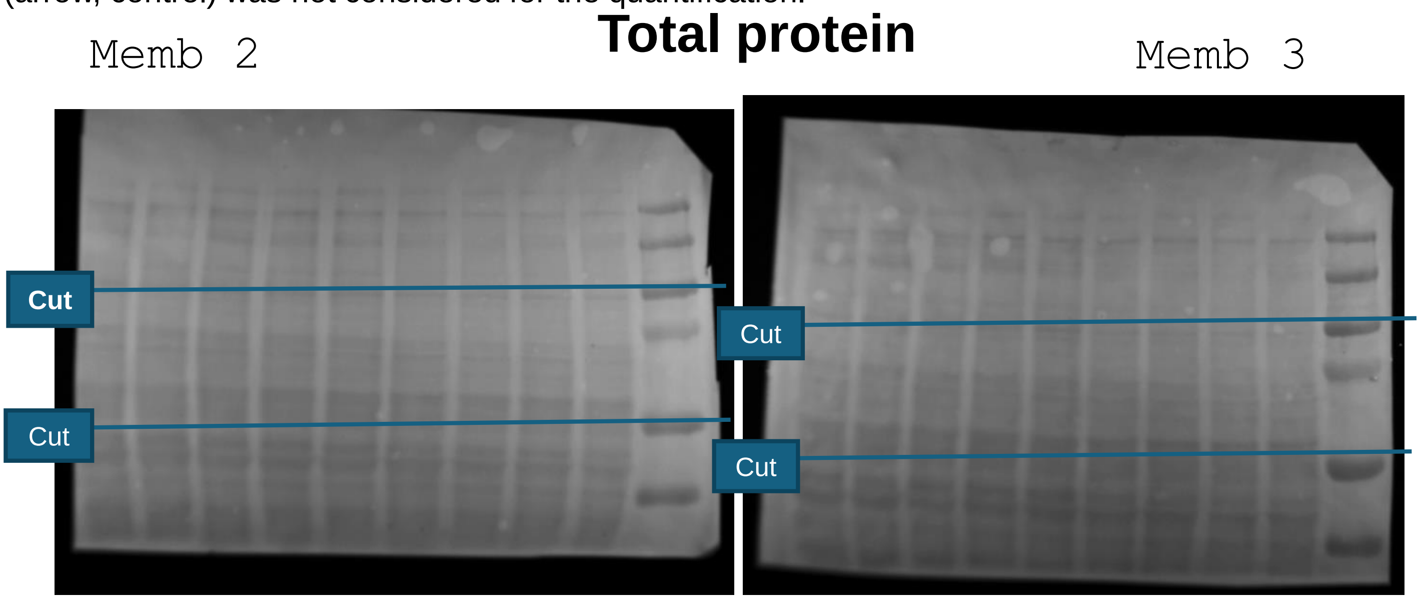

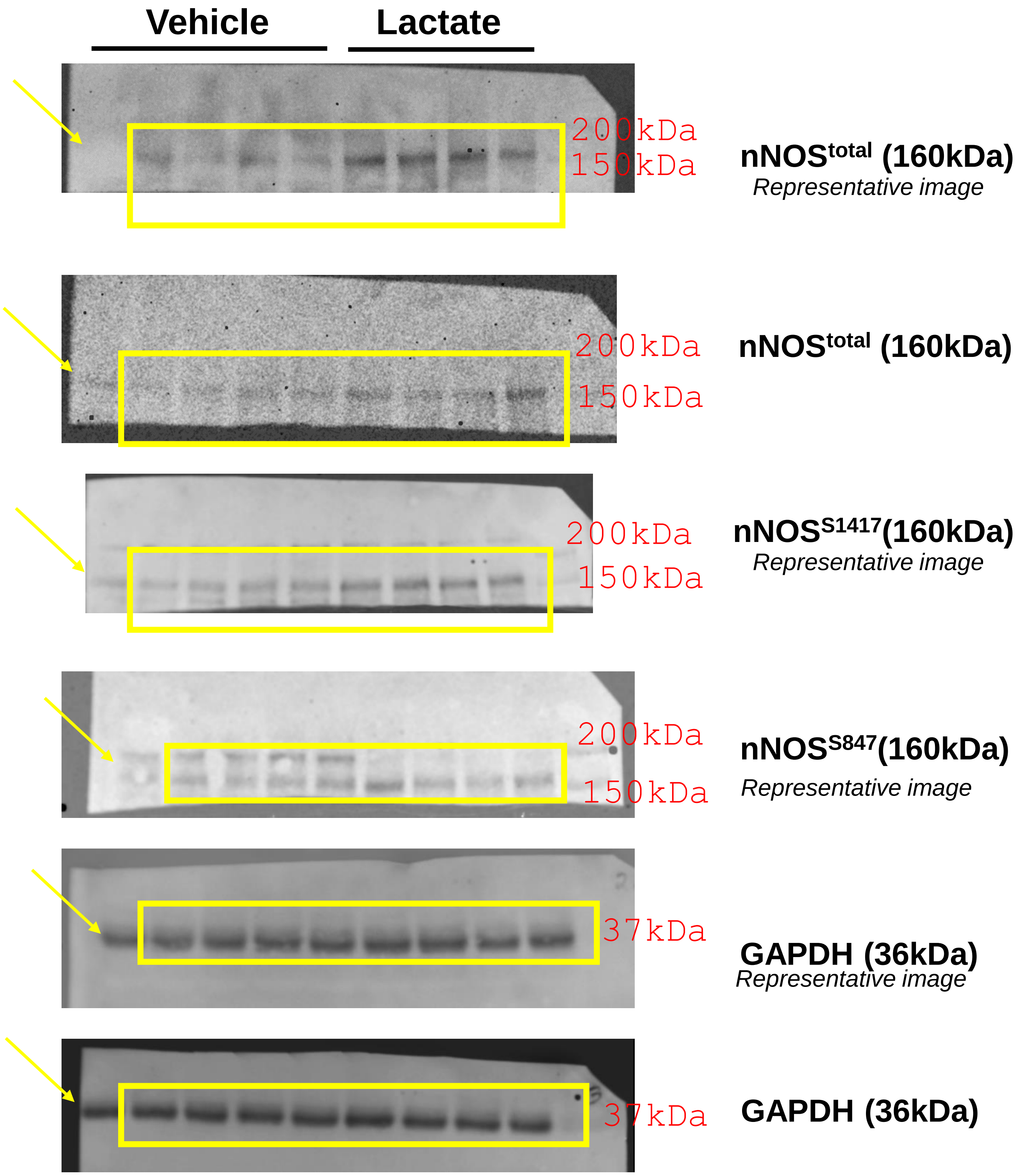

Suppl. Fig 2. The images below are the original blots referenced in Figure 6A. Please note that the first sample (arrow, control) was not considered for the quantification.

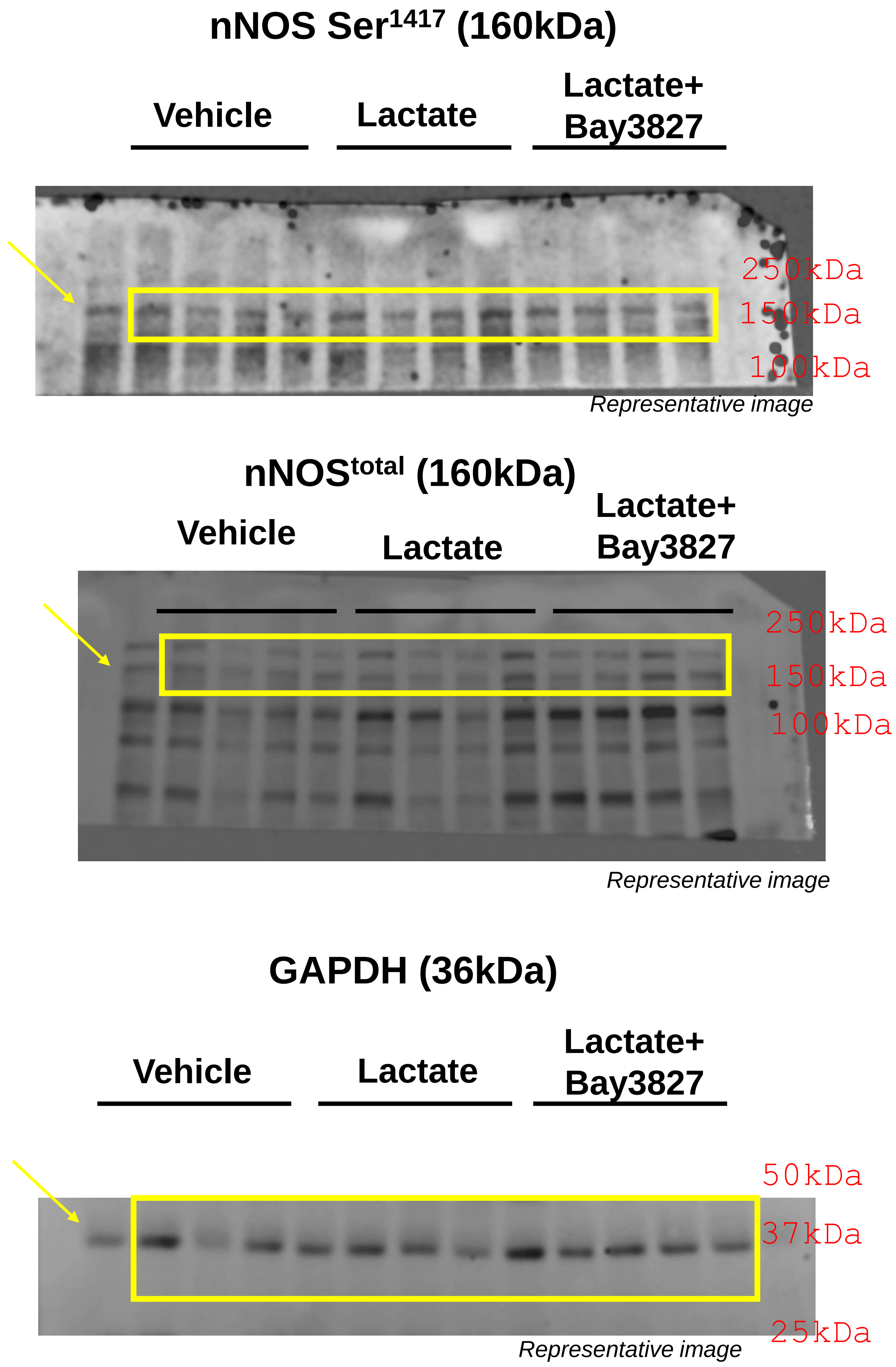

Suppl. Fig 3. The images below are the original blots referenced in Figure 6F.

AMPK (63 kDa)

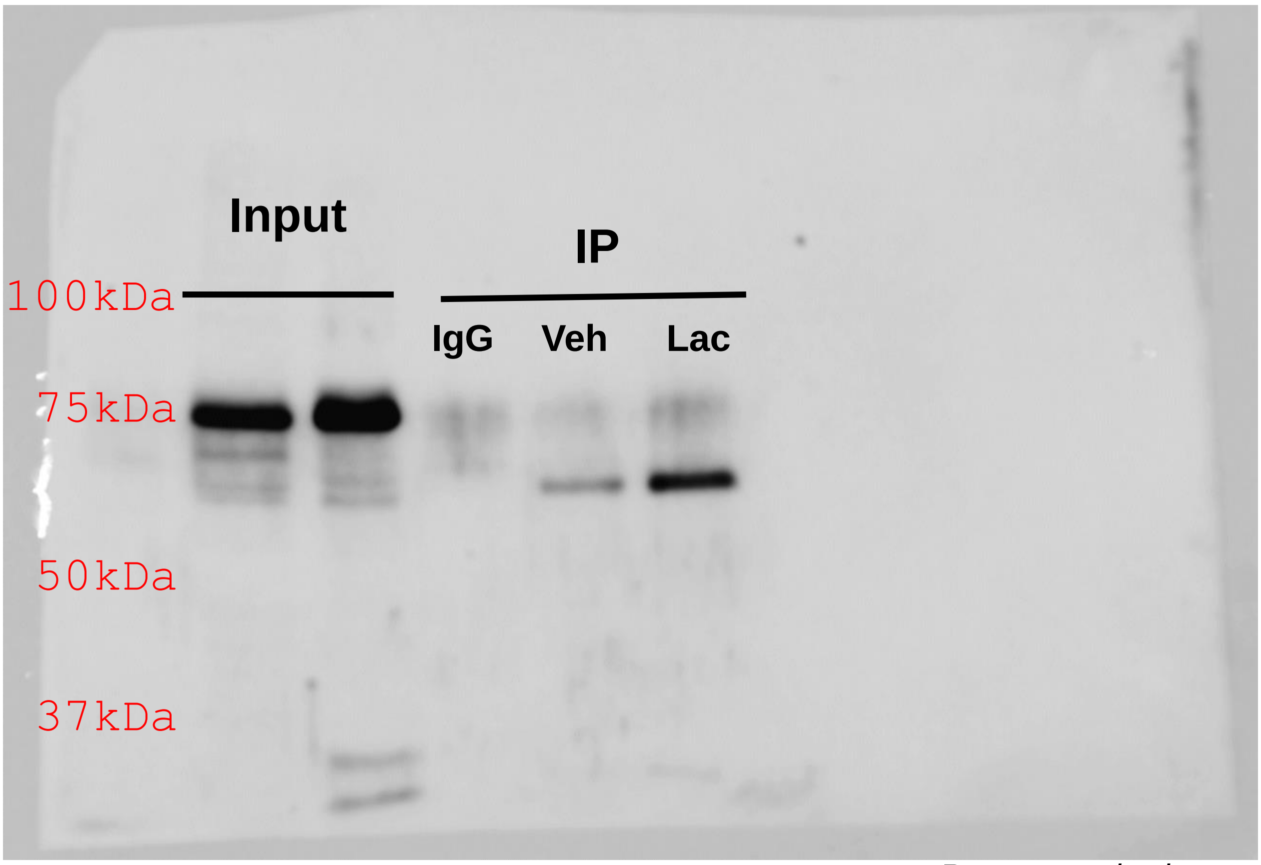

Representative image

Suppl. Fig 4. The images below are the original blots referenced in Figure 7B.

nNOS^{total} (160kDa)

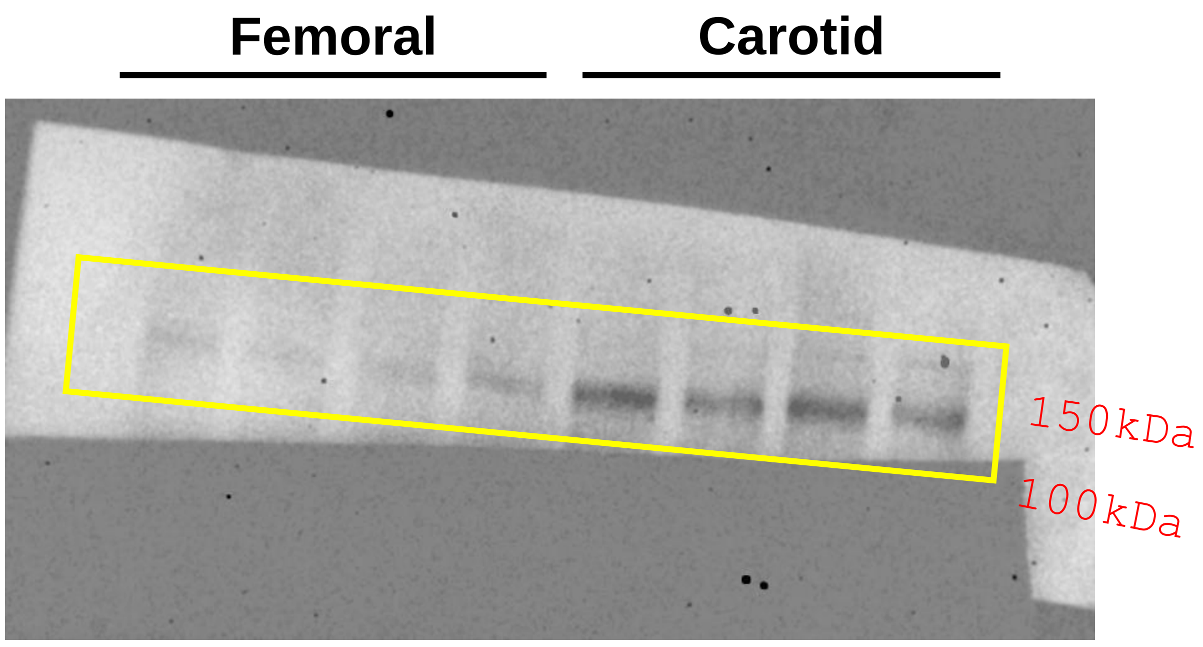

Representative image

GAPDH (36kDa)

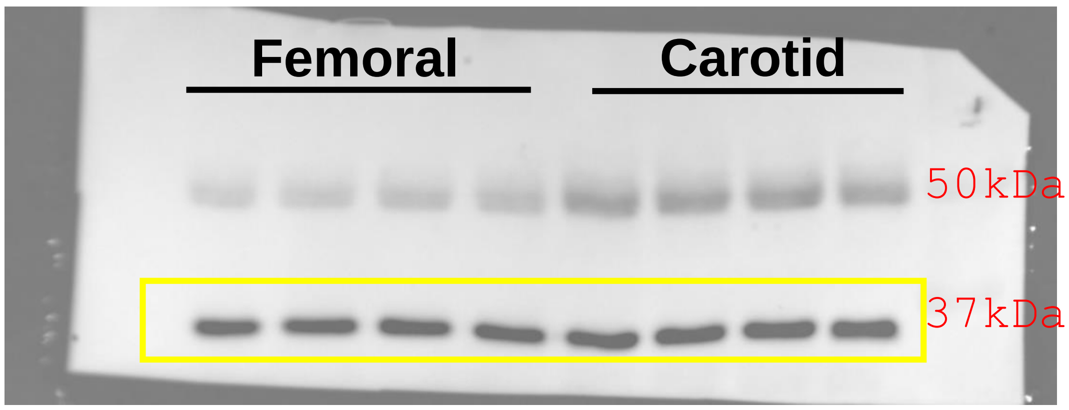

Representative image

Total protein

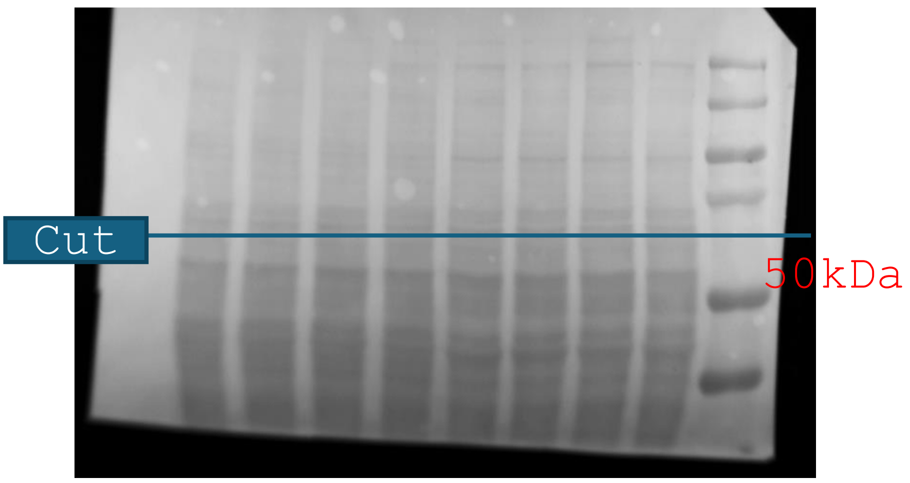

Suppl. Fig 5. The images below are the original blots referenced in Figure 7C.

MnSOD (25kDa)

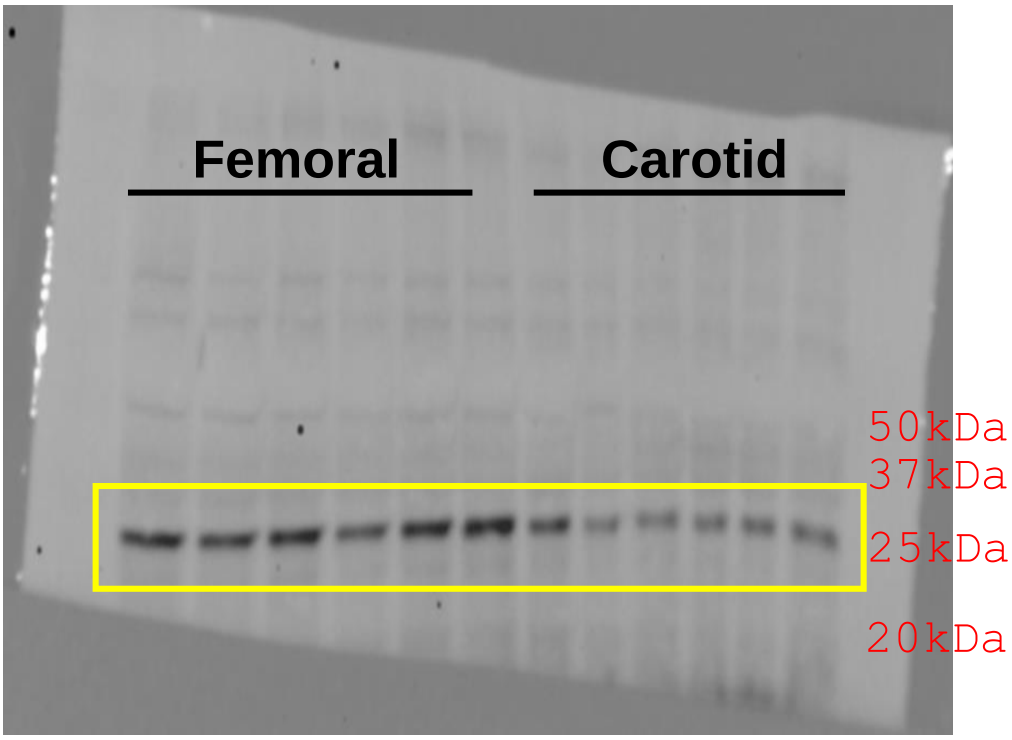

Representative image

GAPDH (36kDa)

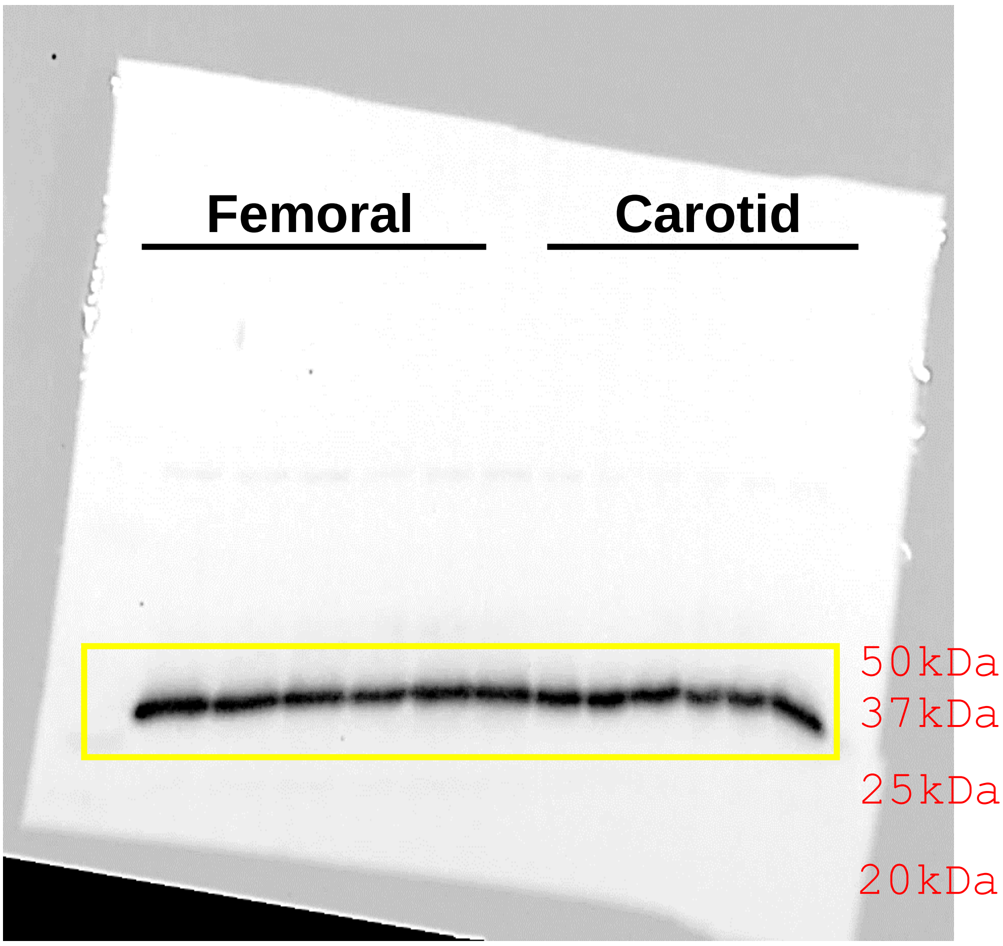

Representative image

Suppl. Fig 6. The images below are the original blots referenced in Figure 7D.

SOD3 35 kDa

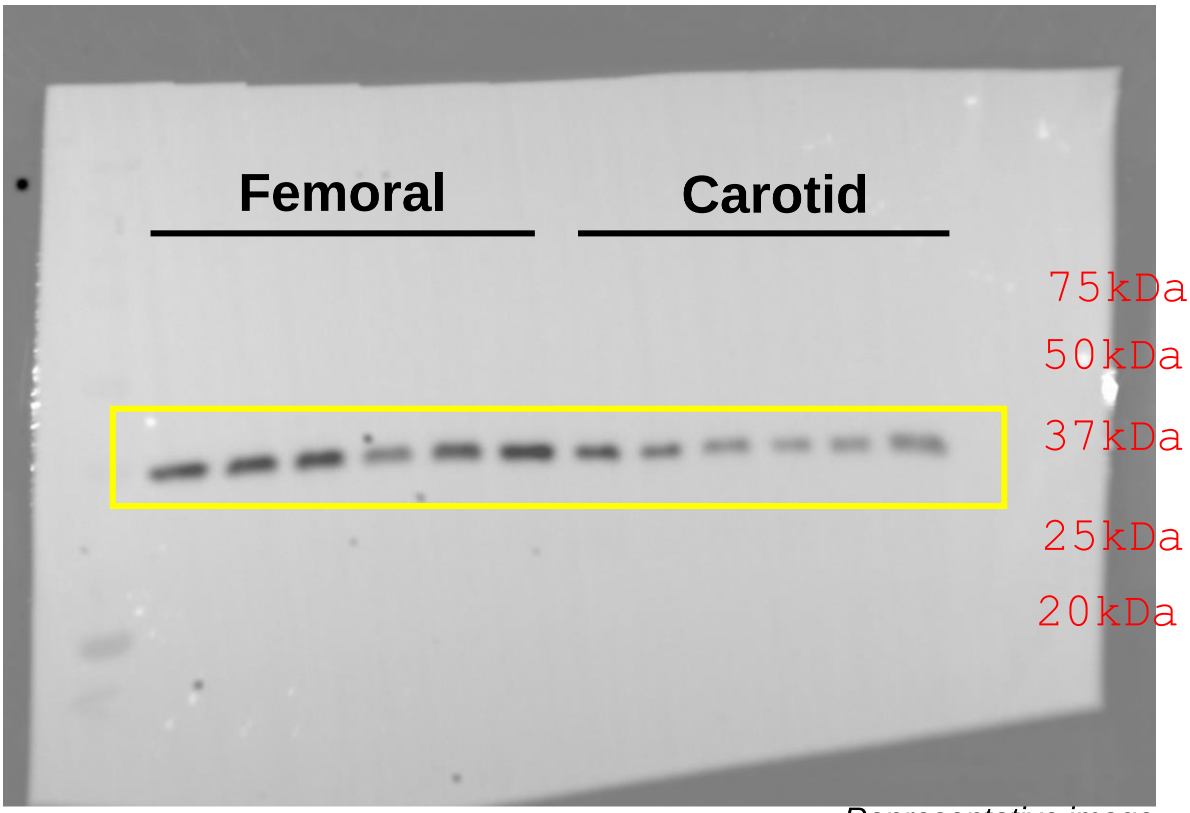

Representative image

GAPDH (38kDa)

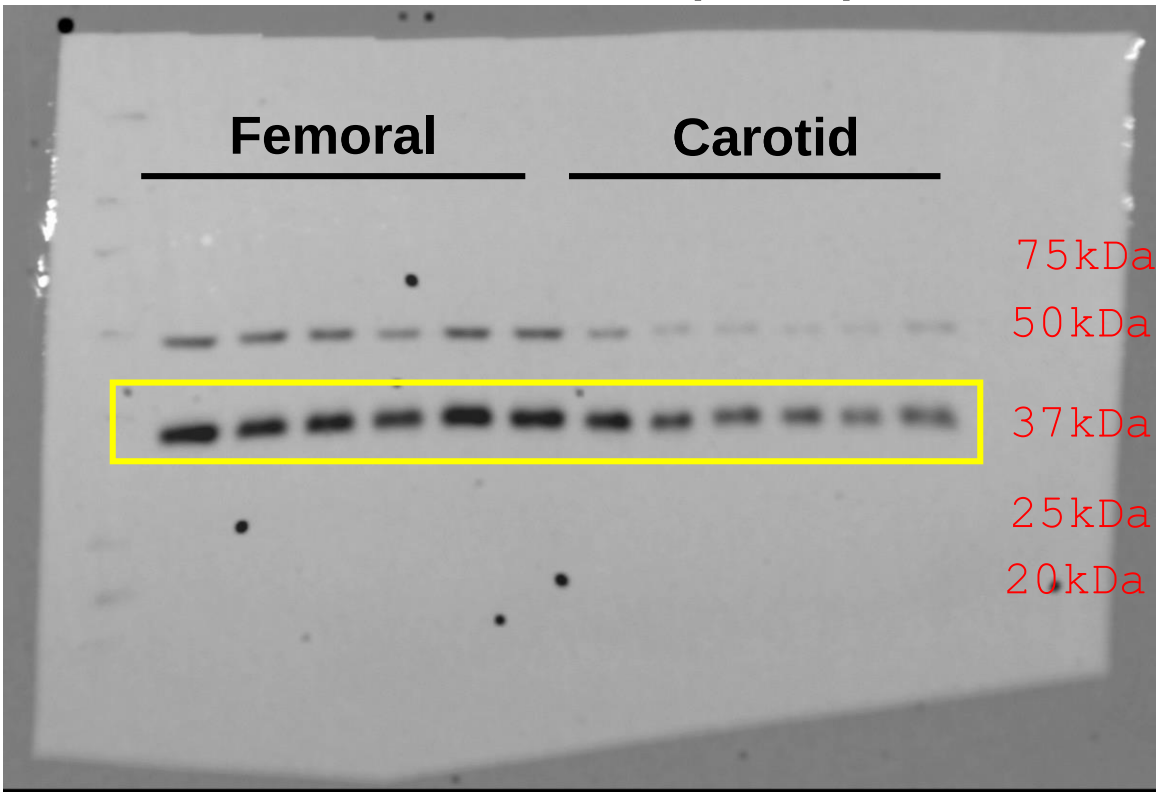

Representative image
